# Data storage architectures to accelerate chemical discovery: data accessibility for individual laboratories and the community

**DOI:** 10.1039/d2sc05142g

**Published:** 2022-11-08

**Authors:** Rebekah Duke, Vinayak Bhat, Chad Risko

**Affiliations:** Department of Chemistry & Center for Applied Energy Research, University of Kentucky Lexington 40506 Kentucky USA chad.risko@uky.edu

## Abstract

As buzzwords like “big data,” “machine learning,” and “high-throughput” expand through chemistry, chemists need to consider more than ever their data storage, data management, and data accessibility, whether in their own laboratories or with the broader community. While it is commonplace for chemists to use spreadsheets for data storage and analysis, a move towards database architectures ensures that the data can be more readily findable, accessible, interoperable, and reusable (FAIR). However, making this move has several challenges for those with limited-to-no knowledge of computer programming and databases. This Perspective presents basics of data management using databases with a focus on chemical data. We overview database fundamentals by exploring benefits of database use, introducing terminology, and establishing database design principles. We then detail the extract, transform, and load process for database construction, which includes an overview of data parsing and database architectures, spanning Standard Query Language (SQL) and No-SQL structures. We close by cataloging overarching challenges in database design. This Perspective is accompanied by an interactive demonstration available at https://github.com/D3TaLES/databases_demo. We do all of this within the context of chemical data with the aim of equipping chemists with the knowledge and skills to store, manage, and share their data while abiding by FAIR principles.

## Abstract 2

With the increasing emphasis on data sharing, reproducibility, and replicability, big-data analytics, and machine learning, chemists must consider database management systems for their laboratory's data storage, management, and accessibility.

## Graphical abstract

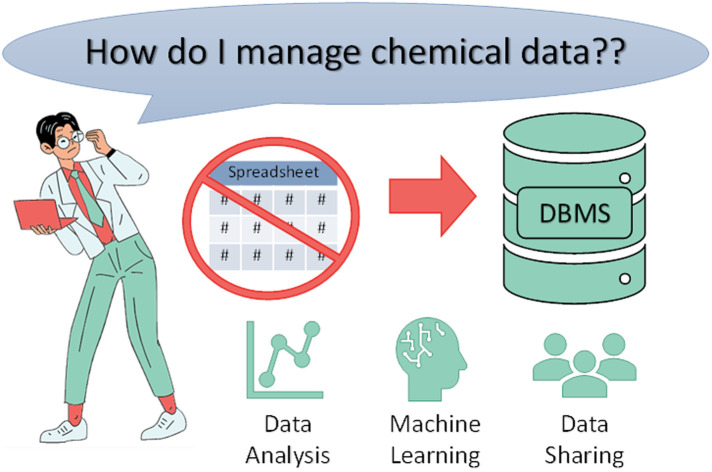

## Introduction

Chemistry is no stranger to big data. As early as the 19th century, chemists compiled atomic and molecular information in catalogs, such as the Beilstein Handbook of Organic Chemistry^1^ and Gmelin Handbook of Inorganic Chemistry,^2^ where molecular, physical, and spectroscopic properties and synthesis pathways were recorded. Journals and periodicals also cataloged the emerging chemical literature with card index systems.^3^ During the next century, more collections of chemical data arose such as the Chemical Rubber Company (CRC) Handbook, which was compiled and sold by a young engineering student trying to pay his way through college.^4^ Eventually, organizations like the International Union of Pure and Applied Chemistry (IUPAC) collected and standardized chemical data, resulting in the Color Books.^5^ With the advent of computer technology and virtual storage in the late 20th century, these catalogs and journals migrated to electronic formats. Today, chemists access big data daily by exploring the literature with resources such as the Web of Science or by searching online chemical catalogs such as SciFinder and Reaxys (which includes the original Gmelin and Beilstein data).^6,7^ The big chemical data in these online formats inform and direct research across the discipline.

With more precise and efficient instrumentation, individual laboratories now generate data on scales previously seen only in these corporate catalogs and databases. For example, a single X-ray crystallography experiment can generate up to 90 gigabytes of data, meaning experiments could generate a terabyte of data in only a few days,^8^ while a molecular dynamics simulation with 100 million atoms can produce 5 gigabytes of data per frame.^9^ These vast catalogs of data are now paving the way for data-driven research methods, offering a move away from time-consuming and resource-expensive Edisonian trial-and-error approaches.^10,11^ Agrawal and Choudhary termed the use of big data in chemistry the “4th paradigm” in chemical research, following the paradigms of empirical science, model-based theoretical science, and computational science.^12^ The shift towards big data-driven chemistry has the potential to amplify lab productivity and escalate scientific progress as much as it has done in the fields of biology and medicine.^13,14^

The generation of large volumes of data and increasing emphasis on data accessibility requires individual laboratories to consider new strategies for data management, as reflected in the growing demand for data management plans by federal funding agencies.^15–20^ Additionally, to effectively create and implement data-driven research methods, there is a need for the data to meet several criteria. A specific data piece, perhaps a spectroscopic measurement for a chemical system, should be findable with a straightforward search. Concurrently, the measurement data should be accessible *via* standard data access procedures, even if the access includes authentication. The data structure and terminology (for instance, the name of the chemical system and the organization of chemical descriptors) should be interpretable by anyone with sufficient domain knowledge. Finally, there should be enough informational data describing the measurement of the chemical system (metadata) that the measurement can be reproduced, making the data reusable. These characteristics—findable, accessible, interoperable, and reusable—constitute the FAIR data principles.^21^ FAIR data principles offer the potential to dramatically enhance data and machine-driven evolutions in chemistry, but it demands not only digitizing chemical data but also capturing the necessary input parameters, process operations, and output data.

Standard data management tools such as spreadsheets and filesystems are not equipped to manage the volumes of data that researchers can now produce, and they are difficult to adapt to FAIR data principles. Most spreadsheet software cannot host more than a million data entries, and these data are maintained at significantly reduced processing speeds; optimum performance is seen with only a few hundred thousand data entries.^22^ Problems pertaining to performance become exaggerated when dealing with multi-dimensional data. Additionally, file-based systems facilitate redundancies, which increase storage costs and enable data inconsistencies. The embedded auto-correct features in many spreadsheets have also notoriously caused data errors in published data.^23,24^ The system of spreadsheets, filesystems, and laboratory notebooks alone will not meet the needs of chemists to store and share growing amounts of FAIR data.^15,25–27^

Database management systems (DBMS) provide solutions to many of these problems. Databases store large quantities of similar, often multi-dimensional data in a consistent organizational structure that can abide by FAIR. Databases are readily scalable, searchable, and sharable. Additionally, as data analyses (specifically, big-data analyses) become a more integral part of chemical discovery, chemists will need time-saving tools to automate these processes. A database's search infrastructure and consistent organizational structure can accelerate and enable automated analyses. Further, databases are critical for (semi)autonomous robotic experiments, as they allow for the management of large data volumes and automated analyses.^28^

Domain specific databases have arisen to store FAIR data, such as the Materials Project^29^ for inorganic materials, the Cambridge Structural Database (CSD)^30^ for crystal structures, and the Protein Data Bank (PDB)^31^ for protein structures. However, for a chemist interested in creating databases for their specific chemical domain or in their own laboratory, the educational resources can be complex. Hence, there is a need to provide information to train chemists to manage large data with databases that abide by FAIR data principles.

In this Perspective, we aim to present an introduction to database fundamentals for a chemistry audience. We first illustrate the advantages of databases over standard file-based data management before describing basic terminology and database design principles. We explore data parsing along with Standard Query Language (SQL) and No-SQL database architectures by exploring the extract, transform, and load process for building a database. Finally, we reflect on some overarching challenges in database design. We do all this with chemistry-specific examples and explanations to promote the creation and accessibility of domain specific data in the realms of FAIR data. We also provide a collection of interactive examples to complement the discussion in this article, which can be accessed at https://github.com/D3TaLES/databases_demo.

## Database fundamentals

### Why databases?

In modern chemistry, the spreadsheet is a ubiquitous tool for storing and analyzing data. The spreadsheet can be effective for managing and analyzing a few to thousands of datapoints, especially when users are familiar with the tools and data formats. Given the ease and ubiquity of spreadsheet-based systems, why should one invest the time and effort to build and learn a DBMS?

**Fig. 1:**
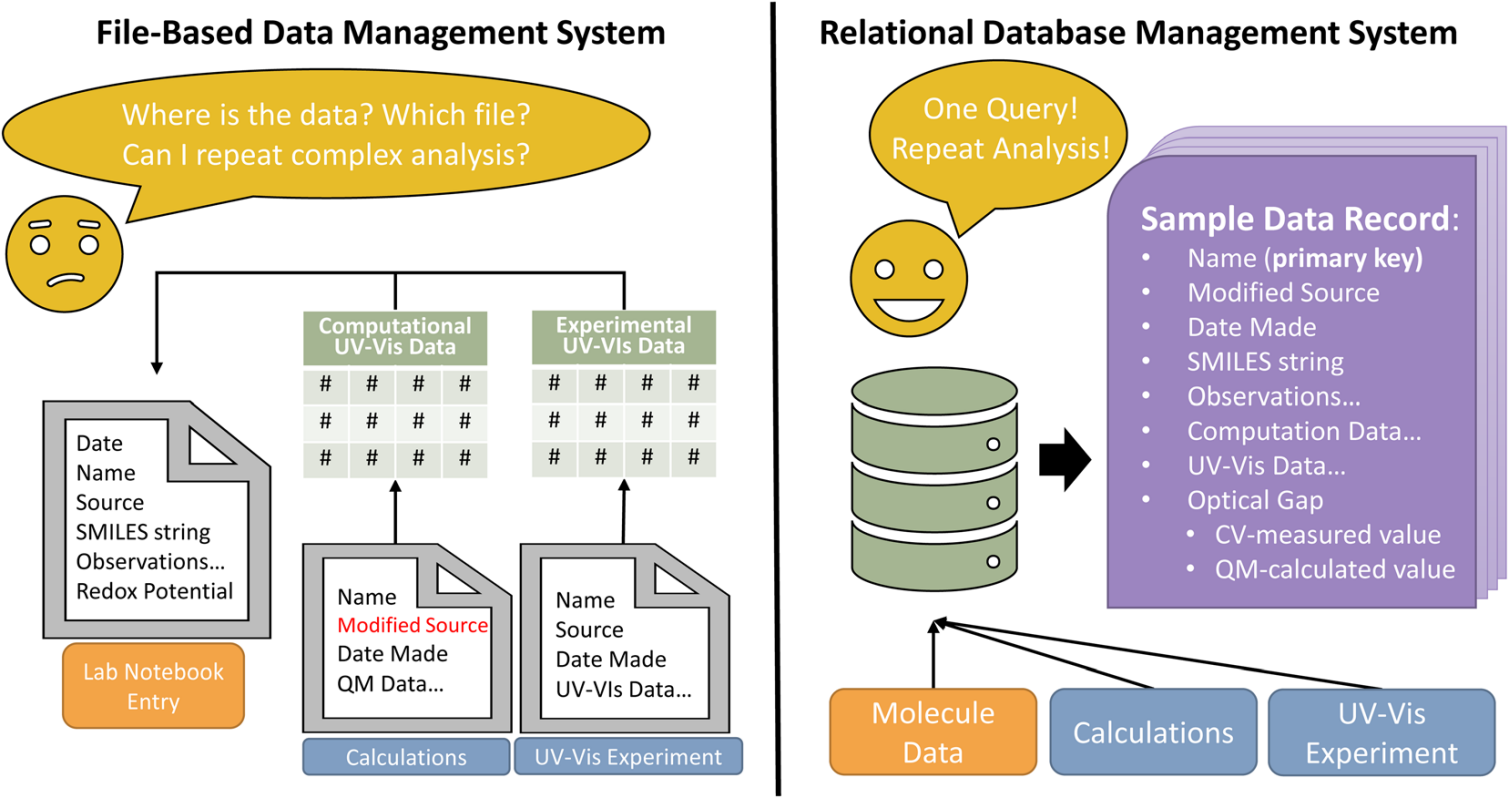
Schematic demonstrating advantages of a (right) database management system (DBMS) over a (left) file-based data management system. Note some advantages of a DBMS over file-based data management: consolidated data, fast and repeatable data queries that replace complex data transformations, and reduced risk of redundant and inconsistent data.

Scientific data generated in research laboratories are saved across several files with diverse formats. This data heterogeneity impedes rapid analysis when tools such as laboratory notebooks and spreadsheets are used to store processed data. To demonstrate the utility of databases compared to lab notebooks and spreadsheets, consider the problem of comparing singlet excitation energies (or wavelengths) determined *via* a quantum-chemical calculation with the optical response measured in a UV-Vis absorption experiment (Fig. 1). First, a researcher opens the output file from the quantum-chemistry software, extracts the desired energy values and stores them, perhaps in a spreadsheet. The researcher must then extract and store data points from the absorption spectrum, plot the spectrum, and identify the absorption energies.^32^ Even if the data extraction process is automated with code, the researcher must manually transfer the data to a laboratory notebook or another spreadsheet to compare the DFT-computed and experimentally-measured energies. Often, data are manually transferred again to another specialized software for analysis, and this entire process must be repeated for each additional experiment. The process is clunky and time-consuming to repeat. Sharing data introduces more problems because raw data and calculations may be in multiple spreadsheets, which may not be readily interpretable by collaborators, and sharing files *via* email or even some file sharing apps can create issues with version consistency. When using a database, raw data are imported directly into the database once. All subsequent analysis, calculations, and comparisons find and use data from the database. Additionally, database access can be granted to collaborators, enabling easy and constantly up-to-date data sharing.

Consider another problem: imagine plotting the data from a series of UV-Vis experiments with benzene and like derivatives (*e.g.*, nitrobenzene, anthracene) where only molecules with a singlet excitation greater than 4 eV are plotted. Here, the researcher must first extract data from the raw experimental and computation data files. There are multiple ways to arrange this data in the spreadsheet; we consider the following – columns for absorbance (transmittance), absorption wavelength, excitation energy, and the molecule identifier. Within a spreadsheet, plotting spectra only when the excitations are greater than 4 eV requires manual selection or sophisticated data transformations. To perform this analysis on another set of experiments, the researcher must repeat this entire process. Alternatively, for data stored in a database, a single line of code fetches the data, and a few lines of code plot the analysis. Because databases embed relationships between like data, a minor modification to the original query would perform the analysis on any new data. The advantages from these examples are demonstrated in the accompanying code.^33^

As shown by this thought experiment, the use of databases to store data can promote rapid analyses. Furthermore, databases are designed to manage large quantities of data and are easily adapted for big data analysis. The sections that follow detail the processes of inserting computational and UV-Vis data into a database and making queries like the ones discussed above. They also discuss the distinct types of databases that can be used along with the pertinent terminology.

### Database terminology

Before delving deeper into database structure and design, we must first establish a basic terminology (Table 1). A database is a collection of data structured in a manner that captures the relationships between groups of like data. These individual pieces of data are termed data records. For example, a database may contain a group of data concerning molecules with UV-Vis and computational data. A single data record may correspond to a single molecule. Each data record has a series of attributes that contain information about the record. So, each molecular data record in our example might have attributes such as source, date synthesized, and UV-Vis data. One attribute must uniquely identify the record; this becomes the primary key. The primary key that identifies a molecule data record might be the molecule IUPAC name or a SMILES^34^ or SELFIES^35^ string (Fig. 1). Similar data records are grouped together so that each data grouping has a defined organizational structure. The organizational outline of a data group is a schema, a map that notes how each attribute in a data record is related. By definition, all records in a data group use the same schema. Defining the schema is critical for efficient database searches and constructing FAIR data; schema will be discussed in detail later.

**Table 1:** Database terminology definitions, as adapted in part from Principles of Database Management^36^

Term	Definition
Database management system (DBMS)	A software package consisting of several software modules used to define, create, use, and maintain a database.^36^
Database	A collection of related data items within a specific process or problem setting stored on a computer system through the organization and management of a database management system.^36^
Data groups	A collection of related data that is stored in the same format.
	SQL term: **Table**, No-SQL term: **Collection**^37^
Data record	A complete data instance for an individual item. A data group contains many data records, each containing different data in the same structure.
	SQL term: **Row**, No-SQL term: **Document**^37^
Attribute	A characteristic of a data record.
	SQL term: **Column**, No-SQL term: **Field**^37^
Multidimensional data	Data where an attribute is more than a single item. For example, an attribute may be a list, or it may include sub-attributes. This requires special data structures. In SQL, multidimensional data are handled with **Table Joins**, while in No-SQL, they are handled with **Embedded Documents**.
Primary key	A selected candidate key that identifies tuples in the relation and is used to establish connections to other relations; must be unique within the relation.^36^
Schema	The description of the database data at different levels of detail, specifying the data items, their characteristics and relationships, constraints, *etc.*^36^
Query	The request and retrieval of data from a database.
Insertion	The addition of data to a database.
Extract transform load (ETL)	The process in which data are extracted (E) from the source systems, transformed (T) to fit the database schema, and then loaded (L) into the database.^36^

## Building a database: extract, transform, and load

**Fig. 2:**
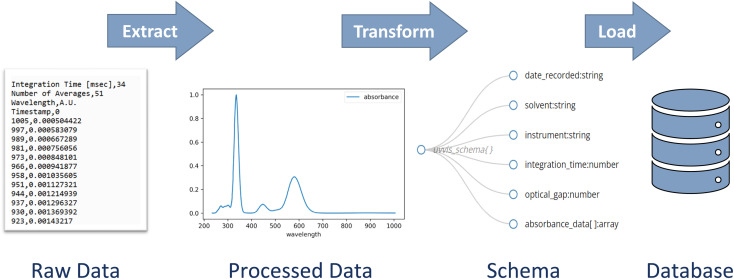
Schematic depictions of fundamental steps in populating a databaes – extract, transform, and load – depicted here in the context of UV-vis spectroscopy data.

Building a database and populating it with data involves three key steps: extract, transform, and load (ETL) (Fig. 2).^38^ Data must first be extracted from the raw data files, and then transformed into a structure that is compatible with the database schema. Finally, the transformed data must be loaded into the database. The development of this process is a critical step toward efficiently populating a database.

### Extract

The process of extracting data from the original files is the step most like the manual processes used in file-based data management systems. In fact, data extraction for a database can be done manually by opening a data file and identifying key data. For example, in the UV-Vis optical absorption example above, the researcher could open the UV-Vis spectrometer output file, identify a particular absorption peak, and input that value into the database.

**Fig. 3:**
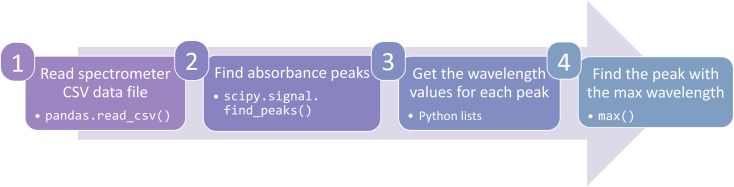
Schematic describing the four lines of code needed to extract data from a spectrometry comma-separated values (CSV) file and determine the energy of the first low-lying excited state related absorption peak. The bullet points note the python command needed for each line. Full code can be found in the accompanying demonstration code.

However, extraction can be automated with code to expedite data analysis and reduce human error. There are many open-source packages that reduce the amount of effort to write parsing code. Imagine our researcher's spectrometer produces a spreadsheet with wavelength and absorbance data. A mere four lines of code in the coding language Python with the packages *pandas* and *scipy* could extract data and find the minimum absorption energy (Fig. 3). Moreover, those four lines of code are applicable to all future spectrometer data files. A more in-depth discussion of parsing techniques is beyond the scope of this Perspective, but a full demonstration can be found in the accompanying code.^33^ Regardless of the method used, extraction should pull important data from the raw data files so it will be ready for the next step.

### Transform

After extraction, data is transformed into the schema-specified format. Schema design is the first step in constructing a database. Designing the database schema is similar in concept to planning the rows and columns in a spreadsheet. Advanced spreadsheet users know that, especially when dealing with multi-dimensional data, deliberately planning the column/row structure alleviates many difficulties later during analysis. While it can be time consuming on the frontend, appropriate schema design is essential for an efficient and FAIR database. Unintuitive schema design yields non-interoperable data. Additionally, because database searches use schema structure, inefficient schema design can produce time-intensive queries. For example, with a database of computational and absorption data, a small molecule chemist might be most likely to query small molecules and their properties, so the molecule-centric schema (where each data record is a molecule) would be most efficient for the laboratory. On the other hand, a computational chemist might more often query individual calculations, so a computation-centric schema (where each data record is a computation) would be most effective for that laboratory.

**Fig. 4:**
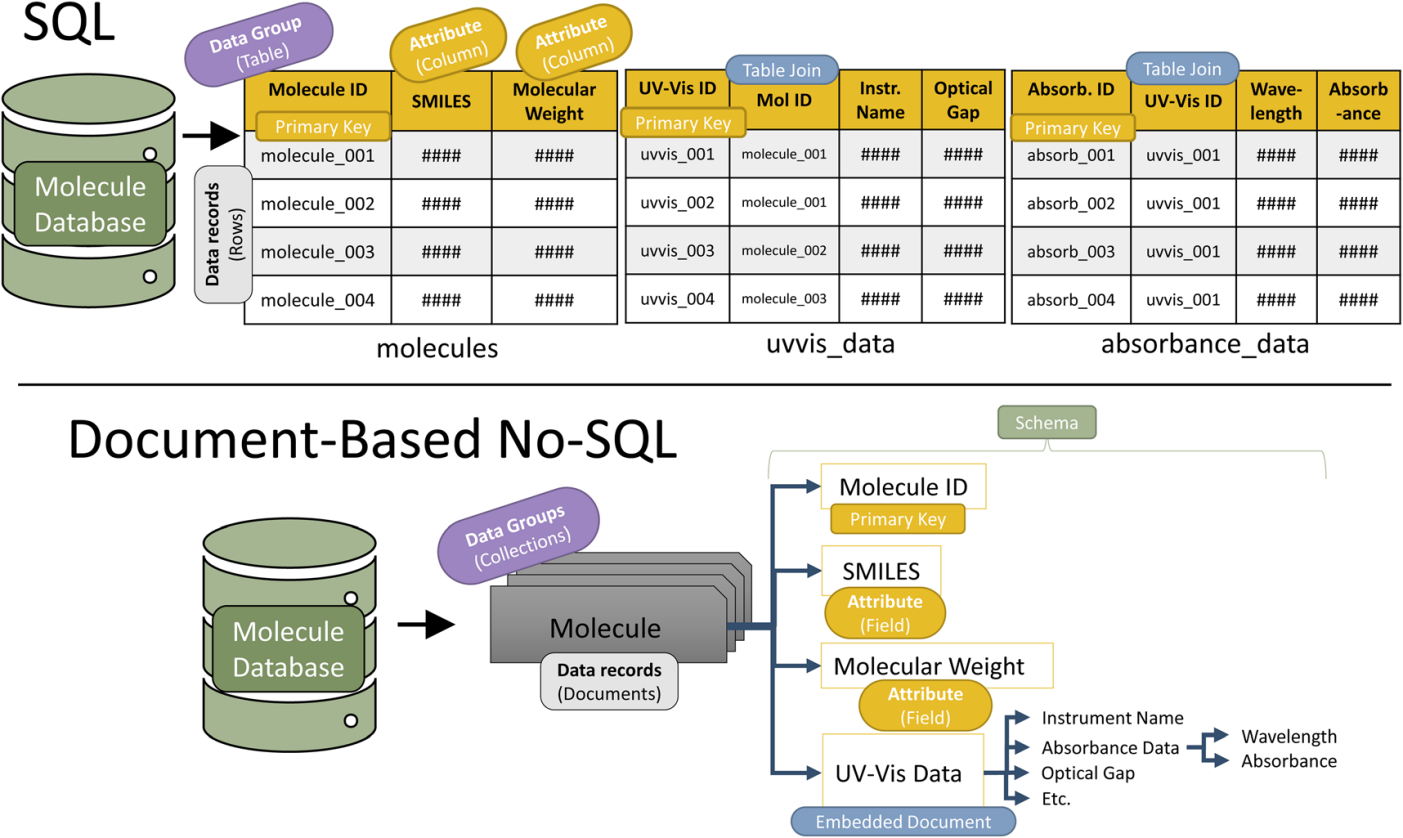
Schematic representation of the two most common schema types in databaes design (SQL and No-SQL) depicted in the context of UV-vis spectroscopy data.

The first decision in schema design is the schema structure type. The two most common structure types are structured query language (SQL) and No-SQL (Fig. 4).^37^ SQL is structured like a series of tables, while No-SQL is structured like a branching tree. Both structure types have a master schema (organizational structure) that all records must follow. Additionally, in both types, one of the attributes for each record must be a uniquely identifying primary key. This enables records to be identified and easily searched. Often, primary keys are randomly generated strings of numbers and letters. For example, the digital object identifier (DOI) generated for every published article frequently serves as a database primary key.

#### SQL

The SQL database structure is the original data management structure. It contains collections of two-dimensional tables, akin to collections of spreadsheets pages. The table rows are data records, and columns are attributes. Each attribute (column) can contain only a single numerical or text value for each record (row). When a data record has an embedded attribute, an SQL database uses multiple tables. For example, a molecule may contain the attribute *UVVis_Data*; however, *UVVis_Data* contains embedded attributes such as *Instrument_Name* and *Optical_Gap*. To accommodate these data, the first table contains the molecule record with its primary key and its regular attributes, while another table contains *UVVis_Data* and its attributes. Each record in *UVVis_Data* connects to the molecules table with a table-joining column. This column contains a molecule primary key (Fig. 4).^39,40^ There are several advantages to an SQL structure. A well-implemented table structure eliminates many data redundancies, increasing data storage efficiency. Also, because of its longevity, the SQL data structure is well documented and supported. These databases can be well-secured, and all SQL-structured databases use the universal Standard Query Language (SQL, from which these databases derive their name).

#### No-SQL

No-SQL structures contain one or more collections of records (called a document in many types of No-SQL). Within a collection, all documents share a schema. Schemas have a tree-branch structure. Each document contains a series of attributes (branches in the tree), each of which may contain a value or list. An attribute may also contain embedded attributes, *e.g.*, smaller branches off the main branch. Fig. 4 shows the nested nature of a No-SQL schema for the *UVVis_Data*. These nested attributes provide scalable depth to a No-SQL database. A single document can easily hold all related data for a record like a molecule, simplifying schema interpretation. Additionally, a No-SQL schema is flexible. This enables dynamic schema adjustments amid the development processes and allows the shaping of schemas to fit expected queries, making future data transactions extremely efficient.^37^ Finally, the modular format of documents allows these databases to be scaled to multiple servers.^41^ A portion of the documents can easily be transferred to a new server if the original runs out of space.

#### Selecting a schema

Both SQL and No-SQL schema types have advantages and disadvantages. For instance, the strict table-based SQL structure limits schema design options. An application's data must conform to an SQL table schema rather than the other way around. Additionally, the restricted schema structure inhibits a schema designed around Perspective queries, often leading to much slower query times.^37^ The interconnected table structure also prevents divided storage, limiting scalability. On the other hand, unlike SQL, No-SQL databases cannot guarantee perfect consistency between documents because separate documents are more prone to redundancies. These redundancies also make No-SQL databases bigger and less storage-efficient than SQL. Additionally, No-SQL databases do not share the Standard Query Language, so each database software can have its own query format.

Ultimately, No-SQL databases are best for prioritizing flexibility, ease of design, and scalability, while SQL databases are best for prioritizing efficiency and consistency.^42,43^ There are many open access No-SQL software, the most notable of which is MongoDB,^44^ known for its user-friendly interface and high consistency despite the limitations of No-SQL structures.^45–47^ Common SQL software includes MySQL,^48^ PostgreSQL,^49^ and Oracle, though software matters less with SQL databases since they all use the Standard Query Language.^50^ The demonstration GitHub repository for this Perspective gives an example of building a No-SQL database with MongoDB and a SQL database.^33^

Once a schema type is selected, the database designer builds an organizational structure that fits the data needs. The design should be efficient yet intuitive. Fig. 4 depicts an effective schema design for absorption data in both SQL and No-SQL structures. Schema design is by far the most time-intensive aspect of the transform step. Once the schema is designed, data from the extraction step is formatted to match the schema, often done through dictionaries (No-SQL) or tables (SQL).

### Load

Finally, the extracted and transformed data is loaded (or inserted) into the database. Anytime data is written to or read from a database, a transaction occurs. Transactions are the building blocks for database interaction. A transaction that writes information to the database is an insertion, while a transaction that reads information from the database is a query. An insertion or query can be made individually through a single line of code or automated so that hundreds of insertions are performed with one command.

While the ETL process is a critical component in implementing a database, there are other technical considerations involving setting up and managing the database. Such detailed discussions are beyond the scope of this Perspective, but we included a list of external resources with the accompanying examples.^51^ These resources include online tutorials on installing and setting up SQL and No-SQL databases for a variety of operating systems and articles on more abstract data structures for large datasets. Readers may also consult the accompanying interactive databases demonstrations.^33^

## Queries

To access the data in a database, users must interact with it *via* a direct transaction or a user interface called an application programming interface (API). Some database software contain built-in API, and these are often the most effective choice for users new to databases and coding. However, if a user has even minimal coding experience, the easiest way to interact with a database is through a direct transaction. A one-line query can search, filter, and transform data however the user might desire.

**Fig. 5:**
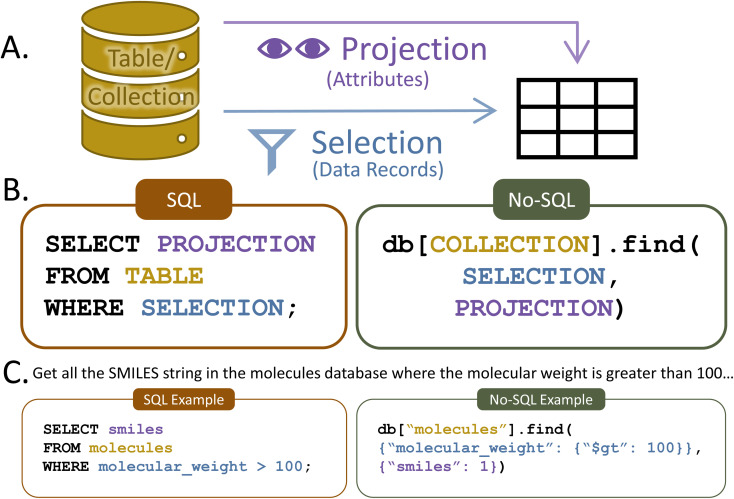
(A) Depiction of the selection and projection components of a database query along with (B) the format of basic queries in SQL-structured and No-SQL-structured databases and (C) example queries in each database type. Note that example No-SQL queries use the MongoDB query format because there is no standard No-SQL query language.

A basic query contains two parts: selection and projection. The selection portion filters the data record(s) (rows for SQL, documents for No-SQL) that will be returned. The projection specifies the record attribute(s) (columns for SQL, fields for No-SQL) that will be shown. For example, imagine a researcher wants to know the SMILES strings^34^ for all molecules in a database that have a molecular weight of more than 100 g mol^−1^. The selection would stipulate only data records with a molecular weight greater than 100 g mol^−1^, while the projection would specify the return of the SMILES attribute (Fig. 5). Alternatively, the researcher might like to list the lowest-lying excited state energy for every molecule or find and count all molecules with more than ten atoms. Basic queries like this are quick and easy in both SQL and No-SQL databases, even when tens of thousands of molecules are present.

**Fig. 6:**
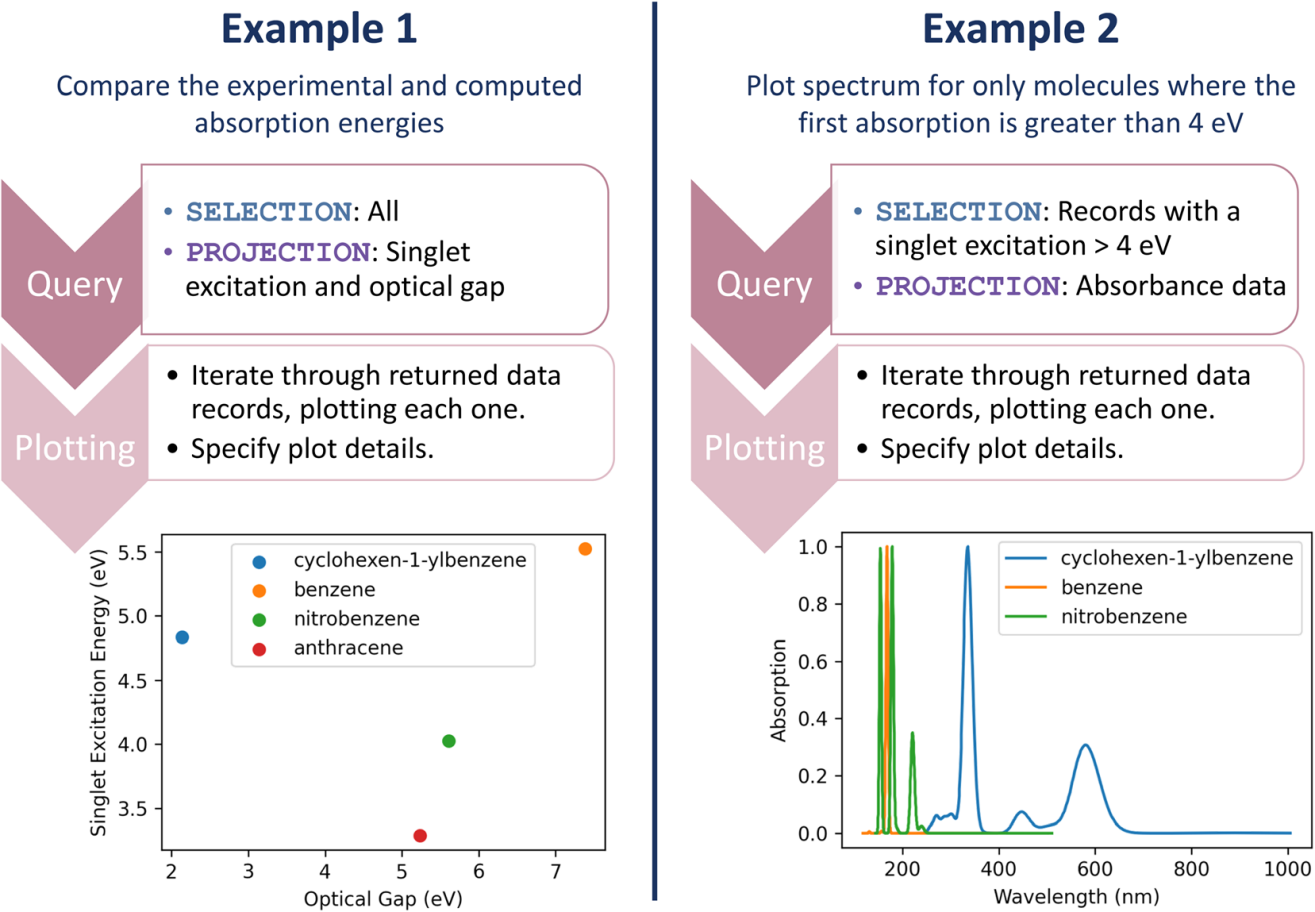
Queries and plots for comparing singlet excitation and experimental optical gap (left) and plotting the spectrum of only molecules where the singlet excitation is greater than 4 eV (right).

Let us return to the multi-faceted analyses involving the UV-Vis first singlet excited state energies and computational modeling: (1) comparing the experimental and computed absorption energies and (2) plotting the absorption spectrum for only molecules where the first absorption is greater than 4 eV. Again, straightforward one-line queries can gather the data for these analyses. Subsequently, a couple of lines of code can produce analysis plots. Fig. 6 demonstrates the query and plotting steps or each of these examples, depicting the resulting plots; full code is available in the accompanying resource.^33^ Most importantly, these queries and plotting are readily repeated. The next time our researcher runs a set of experiments, the entire analysis occurs with the push of a button.

## Database longevity

After database construction, designers must consider database backups. Regular and reliable database backups are essential for an effective database because it is not a matter of if something will go wrong with the database but when. Modern databases are vulnerable to failures ranging from hardware malfunctions to ransomware attacks to human error. But consistent and reliable backups can ward off the potentially catastrophic effects of these failures.

There are four types of database backup: full, incremental, delta, and logs. Successful databases often use all four types. As the name suggests, a full backup duplicates the entire database for storage. While thorough, these backups require significant storage space, often too much to perform more than once every week or two. Incremental backups, on the other hand, duplicate storage for all database records that have changed since the last full backup. Similarly, delta backups record the transactional changes since the last backup of any kind. These three backup techniques constitute most database backup systems. The final backup type is less a backup than an emergency record. Logs are the systematic record of every database transaction. Theoretically, a database can be rebuilt by rerunning every transaction that has occurred from the logs. However, this method is neither dependable nor efficient. Because the log files grow quickly, the log history is frequently wiped clean.

Regardless of the specific backup plan designed, the most important part is redundancy. While database design tries to avoid redundancies, database backup plans should incorporate redundancies wherever possible. Save multiple full backups, saved on multiple servers, ideally on multiple networks.

## Challenges

In 2017, The Minerals, Metals & Materials Society (TMS) issued a report cataloging challenges with building effective materials data infrastructures.^52^ Many challenges centered on the community's minimal understanding of data storage and management options and associated best practices, a problem this Perspective seeks to address.^53^ One of the most high-impact challenges identified was the lack of developed, agreed-upon data schemas. As more domain-specific databases emerge, challenges arise in the interaction of databases with various users and other databases.^54–56^ To effectively share data across labs and data platforms, there must be some degree of agreement between the data representation, terminology, and formats. A key first step in developing universal schemas is educating all members of the scientific community about domain-specific ontologies, which are the fundamental categorization of objects and the definition of relationships between the categories.^57–59^ This will enable scientists' contributions to the philosophical endeavor to develop universal ontologies that can lay the foundation for a universal schema. Yet, to establish standard ontologies, scientists must first engage in their design.

The second challenge in database development is the curation of gathered data. As the adage goes, “garbage in, garbage out.” If inconsistent, incorrect, or outlier data enters a database, the data analytics performed on that data will produce spurious insights. Too often, the best method for data curation remains human gut checks. It is much easier for a human expert than a computer to identify a suspicious peak in an NMR spectrum. At this point, database curation must continue to integrate human data checks to curate incoming data. However, as the quantity of data grows, efforts to automate data curation must follow suit.

The extraction of data from raw data files presents additional challenges. For example, different brands of instruments may produce distinctly formatted output files. Each file will require an individualized parser to extract necessary data. Moreover, some instrument output files do not contain all relevant data, so additional metadata such as molecule concentration, solvent type, and even procedural details must be gathered.

Finally, as more domain-specific databases emerge, challenges will arise in the interaction of databases with various users and other databases. To abide by the FAIR principles, data must be accessible and searchable in an interoperable format.^21^ An API is the most useful tool for human–database interaction. Additionally, to enable data machine-accessibility, databases should incorporate a representational state transfer API (REST API), which presents data for online sharing according to REST internet standards. Unfortunately, if not included in the database software, API and REST API require time and expertise to develop. To circumnavigate these issues, there do exist powerful scientific data-sharing platforms which include API and/or REST API capabilities.^54,55,60–63^

## Conclusions and outlook

As chemistry enters the “fourth paradigm” of scientific discovery, it will be essential to effectively store and manage data. Such efforts will not only enable the use of big data analytics and machine learning but also establish the data management framework needed to integrate robotic/autonomous experimentation into laboratories. While there remain challenges in constructing and maintaining a DBMS, storage efficiency, query speeds, and ability to abide by FAIR data principles are unparalleled in DBMS when compared to file-based systems. Therefore, we encourage all chemistry laboratories to explore DBMS. At a minimum, we encourage laboratories to upload data to existing data databases, such as large-scale repositories and field-specific mid-sized databases, many of which are cataloged in database listings.^54,55,61–67^

Still, the growing data demands of many laboratories will necessitate small-scale laboratory databases. Fortunately, there are many tools available. For those wishing to design a database from the ground up, the software and designs described in this Perspective provide powerful tools for data management. Meanwhile, for those seeking less intensive data management platforms, pre-built data storage structures exist, allowing users to customize a data schema while providing an API and graphical tools for data analysis.^55,68–70^ Ultimately, the transition from file-based data management to DBMS will take many forms across many fields. We hope that the introduction to database terminology and structures provided here will guide chemists through the process of database design.

## Data availability

The data and the code presented in this article are available on the GitHub repository at https://github.com/D3TaLES/databases_demo. This repository contains simple, chemistry-based demonstrations of both an SQL and a No-SQL database structure and experimental file parsing. The repository also contains a list of external resources that give more specific details for setting up a database.

## Conflicts of interest

The authors declare no competing financial interest.

## Author contributions

R. D.: conceptualization, investigation, software, visualization, writing – original draft, writing – review & editing. V. B.: conceptualization, software, writing – review & editing. C. R.: supervision, funding acquisition, writing – review & editing.

## Supplementary Material

## References

[cit1] Luckenbach R. (1981). The Beilstein Handbook of Organic Chemistry: the first hundred years. J. Chem. Inf. Model..

[cit2] Mague J. (1984). Gmelin Handbook of Inorganic Chemistry, Rh.. Organometallics.

[cit3] Barrows F. E. , Investigations of the Chemical Literature, Armour Institute of Technology, New York, 1921

[cit4] Broad W. J. (1979). Rubber Bible Turns 60. Science.

[cit5] Hartshorn R. (2017). Research Data, Big Data, and Chemistry. Chem. Int..

[cit6] Sutton M. (2017). The first chemical database. Chem. World.

[cit7] Mutton T., Ridley D. D. (2019). Understanding Similarities and Differences between Two Prominent Web-Based Chemical Information and Data Retrieval Tools: Comments on Searches for Research Topics, Substances, and Reactions. J. Chem. Educ..

[cit8] Maia F. R. N. C. (2012). The Coherent X-ray Imaging Data Bank. Nat. Methods.

[cit9] Omeltchenko A., Campbell T. J., Kalia R. K., Liu X., Nakano A., Vashishta P. (2000). Scalable I/O of large-scale molecular dynamics simulations: A data-compression algorithm. Comput. Phys. Commun..

[cit10] Glynn C., Rodriguez J. A. (2019). Data-driven challenges and opportunities in crystallography. Emerging Top. Life Sci..

[cit11] Yano J., Gaffney K. J., Gregoire J., Hung L., Ourmazd A., Schrier J., Sethian J. A., Toma F. M. (2022). The case for data science in experimental chemistry: examples and recommendations. Nat. Rev. Chem..

[cit12] Agrawal A., Choudhary A. (2016). Perspective: Materials informatics and big data: Realization of the “fourth paradigm” of science in materials science. APL Mater..

[cit13] Savage N. (2014). Bioinformatics: Big data *versus* the big C. Nature.

[cit14] Hood L., Rowen L. (2013). The human genome project: big science transforms biology and medicine. Genome Med..

[cit15] Jablonka K. M., Patiny L., Smit B. (2022). Making the collective knowledge of chemistry open and machine actionable. Nat. Chem..

[cit16] European Research Council Scientific Council , Open Research Data and Data Management Plans, version 4.1, 2022

[cit17] Huang Y., Cox A. M., Sbaffi L. (2021). Research data management policy and practice in Chinese university libraries. J. Assoc. Inf. Sci. Technol..

[cit18] NIH , Grants Compliance and Oversight, National Institutes of Health, 2022, https://grants.nih.gov/policy/compliance.htm, accessed June 2022

[cit19] Dissemination and Sharing of Research Results – NSF Data Management Plan Requirements, National Science Foundation, 2022, https://www.nsf.gov/bfa/dias/policy/dmp.jsp, accessed June 2022

[cit20] Statement on Digital Data Management, Office of Science, U.S. Department of Energy, https://science.osti.gov/Funding-Opportunities/Digital-Data-Management, accessed October 2022

[cit21] Wilkinson M. D., Dumontier M., Aalbersberg I. J., Appleton G., Axton M., Baak A., Blomberg N., Boiten J.-W., Da Silva Santos L. B., Bourne P. E. (2016). *et al.*, The FAIR Guiding Principles for scientific data management and stewardship. Sci. Data.

[cit22] Excel specifications and limits, Microsoft, https://support.microsoft.com/en-us/office/excel-specifications-and-limits-1672b34d-7043-467e-8e27-269d656771c3, accessed May 2022

[cit23] Ziemann M., Eren Y., El-Osta A. (2016). Gene name errors are widespread in the scientific literature. Genome Biol..

[cit24] Lewis D. (2021). Autocorrect errors in Excel still creating genomics headache. Nature.

[cit25] Howes L. (2019). Chemistry data should be FAIR, proponents say. But getting there will be a long road. Chem. Eng. News.

[cit26] Potthoff J., Tremouilhac P., Hodapp P., Neumair B., Bräse S., Jung N. (2019). Procedures for systematic capture and management of analytical data in academia. Anal. Chim. Acta: X.

[cit27] IUPAC Endorces the Chemistry Go FAIR Manifesto, International Union of Pure and Applied Chemistry, 2019, https://iupac.org/iupac-endorses-the-chemistry-go-fair-manifesto/, accessed July 2022

[cit28] Nisbet M. L., Pendleton I. M., Nolis G. M., Griffith K. J., Schrier J., Cabana J., Norquist A. J., Poeppelmeier K. R. (2020). Machine-Learning-Assisted Synthesis of Polar Racemates. J. Am. Chem. Soc..

[cit29] Jain A., Ong S. P., Hautier G., Chen W., Richards W. D., Dacek S., Cholia S., Gunter D., Skinner D., Ceder G. (2013). *et al.*, Commentary: The Materials Project: A materials genome approach to accelerating materials innovation. APL Mater..

[cit30] Groom C. R., Bruno I. J., Lightfoot M. P., Ward S. C. (2016). The Cambridge Structural Database. Acta Crystallogr., Sect. B: Struct. Sci., Cryst. Eng. Mater..

[cit31] Berman H., Henrick K., Nakamura H. (2003). Announcing the worldwide Protein Data Bank. Nat. Struct. Mol. Biol..

[cit32] Makuła P., Pacia M., Macyk W. (2018). How To Correctly Determine the Band Gap Energy of Modified Semiconductor Photocatalysts Based on UV-Vis Spectra. J. Phys. Chem. Lett..

[cit33] https://github.com/D3TaLES/databases_demo

[cit34] Weininger D. (1988). SMILES, a chemical language and information system. 1. Introduction to methodology and encoding rules. J. Chem. Inf. Model..

[cit35] Krenn M., Häse F., Nigam A., Friederich P., Aspuru-Guzik A. (2020). Self-referencing embedded strings (SELFIES): a 100% robust molecular string representation. Mach. learn.: sci. technol..

[cit36] Lemahieu W. , vanden Broucke S. and Baesens B., Principles of Database Management: The Practical Guide to Storing, Managing and Analyzing Big and Small Data, Cambridge University Press, 2018 10.1089/big.2018.0044 29924650

[cit37] Ali W., Shafique M. U., Majeed M. A., Raza A. (2019). Comparison between SQL and NoSQL Databases and Their Relationship with Big Data Analytics. Asian J. Res. Comput. Sci..

[cit38] Runtuwene J. P. A., Tangkawarow I. R. H. T., Manoppo C. T. M., Salaki R. J. (2017). A Comparative Analysis of Extract, Transformation and Loading (ETL) Process. IOP Conf. Ser.: Mater. Sci. Eng..

[cit39] Goelman D. and Dietrich S. W., A Visual Introduction to Conceptual Database Design for All, in Proceedings of the 49th ACM Technical Symposium on Computer Science Education, 2018-02-21, ACM, 2018

[cit40] Razu Ahmed M., Arifa Khatun M., Asraf Ali M., Sundaraj K. (2018). A literature review on NoSQL database for big data processing. Int. J. Eng. Technol..

[cit41] Cattell R. , Scalable SQL and NoSQL data stores, ACM SIGMOD Record, 2011, 39, ch. 4, pp. 12–27

[cit42] Venkatraman S., Fahd K., Kaspi S., Venkatraman R. (2016). SQL *versus* NoSQL Movement with Big Data Analytics. Int. J. Inf. Technol. comput. sci..

[cit43] Boicea A. , Radulescu F. and Agapin L. I., MongoDB *vs.* Oracle – Database Comparison, in 2012 Third International Conference on Emerging Intelligent Data and Web Technologies, 2012-09-01, IEEE, 2012

[cit44] MongoDB , https://www.mongodb.com/, accessed June 2022

[cit45] Diogo M., Cabral B., Bernardino J. (2019). Consistency Models of NoSQL Databases. Future Internet.

[cit46] Chauhan A. A. (2019). Review on Various Aspects of MongoDb Databases. Int. J. Eng. Res. Sci. Technol..

[cit47] Abramova V. and Bernardino J., NoSQL databases, Proceedings of the International C* Conference on Computer Science and Software Engineering, C3S2E '13, 2013, pp. 14–22

[cit48] MySQL, 2022, https://www.mysql.com/, accessed June 2022

[cit49] PostgreSQL, 2022, https://www.postgresql.org/, accessed July 2022

[cit50] Oracle, 2022, https://www.oracle.com/database/technologies/appdev/sqldeveloper-landing.html, accessed June 2022

[cit51] https://github.com/D3TaLES/databases_demo/blob/main/external_resources.md

[cit52] The Minerals, Metals & Materials Series , Building a Materials Data Infrastructure: Opening New Pathways to Discovery and Innovation in Science and Engineering, TMS, 2017

[cit53] Tanifuji M. , Matsuda A. and Yoshikawa H., Materials Data Platform – a FAIR System for Data-Driven Materials Science, in 2019 8th International Congress on Advanced Applied Informatics (IIAI-AAI), 2019-07-01, IEEE, 2019

[cit54] Blaiszik B., Chard K., Pruyne J., Ananthakrishnan R., Tuecke S., Foster I. (2016). The Materials Data Facility: Data Services to Advance Materials Science Research. JOM.

[cit55] Scheffler M., Draxl C. (2019). The NOMAD laboratory: from data sharing to artificial intelligence. J. Phys. Matter..

[cit56] Himanen L., Geurts A., Foster A. S., Rinke P. (2019). Data-Driven Materials Science: Status, Challenges, and Perspectives. Adv. Sci..

[cit57] Eine B., Jurisch M., Quint W. (2017). Ontology-Based Big Data Management. Systems.

[cit58] Li H. , Armiento R. and Lambrix P., An Ontology for the Materials Design Domain, in Lecture Notes in Computer Science, Springer International Publishing, 2020, pp. 212–227

[cit59] EMMO: an Ontology for Applied Sciences, European Materials Modelling Ontology (EMMO), https://github.com/emmo-repo/EMMO, accessed July 2022

[cit60] Steinbeck C., Koepler O., Bach F., Herres-Pawlis S., Jung N., Liermann J., Neumann S., Razum M., Baldauf C., Biedermann F. (2020). *et al.*, NFDI4Chem – Towards a National Research Data Infrastructure for Chemistry in Germany. RIO.

[cit61] Pizzi G., Cepellotti A., Sabatini R., Marzari N., Kozinsky B. (2016). AiiDA: automated interactive infrastructure and database for computational science. Comput. Mater. Sci..

[cit62] Trisovic A. , Durbin P., Schlatter T., Durand G., Barbosa S., Brooke D. and Crosas M., Advancing Computational Reproducibility in the Dataverse Data Repository Platform, in Proceedings of the 3rd International Workshop on Practical Reproducible Evaluation of Computer Systems, 2020-06-23, ACM, 2020

[cit63] Curtarolo S., Setyawan W., Hart G. L. W., Jahnatek M., Chepulskii R. V., Taylor R. H., Wang S., Xue J., Yang K., Levy O. (2012). *et al.*, AFLOW: An automatic framework for high-throughput materials discovery. Comput. Mater. Sci..

[cit64] Tremouilhac P., Nguyen A., Huang Y.-C., Kotov S., Lütjohann D. S., Hübsch F., Jung N., Bräse S. (2017). Chemotion ELN: an Open Source electronic lab notebook for chemists in academia. J. Cheminf..

[cit65] Frantzen A., Sanders D., Scheidtmann J., Simon U., Maier W. F. (2005). A Flexible Database for Combinatorial and High-Throughput Materials Science. QSAR Comb. Sci..

[cit66] Data Repository Guidance, Springer Nature Limited, https://www.nature.com/sdata/policies/repositories, accessed July 2022

[cit67] Software & Data Resources, Designing Materials to Revolutionize and Engineer our Future (DMREF), https://dmref.org/tools, accessed July 2022

[cit68] Brandt N., Griem L., Herrmann C., Schoof E., Tosato G., Zhao Y., Zschumme P., Selzer M. (2021). Kadi4Mat: A Research Data Infrastructure for Materials Science. Data Sci. J..

[cit69] Yakutovich A. V., Eimre K., Schütt O., Talirz L., Adorf C. S., Andersen C. W., Ditler E., Du D., Passerone D., Smit B. (2021). *et al.*, AiiDAlab – an ecosystem for developing, executing, and sharing scientific workflows. Comput. Mater. Sci..

[cit70] OPTiMaDe, https://www.optimade.org, accessed July 2022

